# Supplementary material for: Genetic analysis defines sites of charge-based interaction between LolA and LolB proteins in lipoprotein trafficking
Source: bioRxiv. 2026 Oct 28:2026.10.27.761055. Preprint. [Version 1] doi: 10.64898/2026.10.27.761055 (PMC13733947; doi:10.64898/2026.10.27.761055)
Supplement: Supplementary file 1 [file NIHPP2026.10.27.761055V1-supplement-1.pdf]

bioRxiv preprint doi: https://doi.org/10.64898/2026.10.27.761055; this version posted October 28, 2026. The copyright holder for this preprint (which was not certified by peer review) is the author/funder, who has granted bioRxiv a license to display the preprint in perpetuity. It is made available under a CC-BY 4.0 International license.

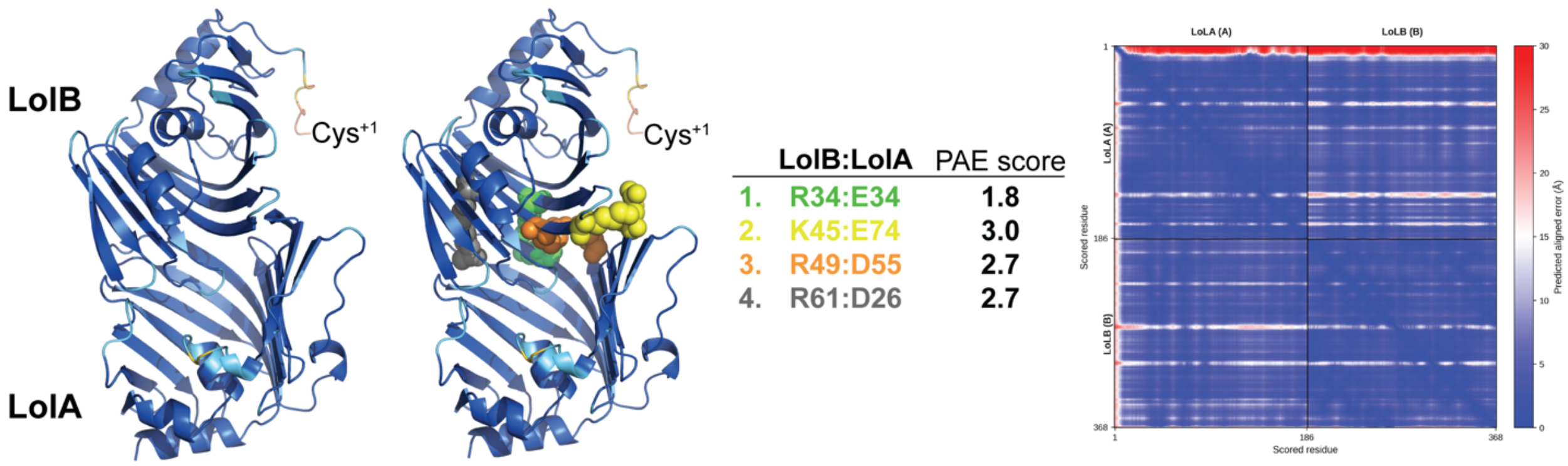

Figure S1. AlphaFold predicted LolB:LolA interaction. Structures are colored by pLDDT values. PAE scores of select predicted interactions are provided. Multimer prediction has a pTM = 0.89.
